# Complete mitochondrial genome and phylogenetic analysis of willow ptarmigan (*Lagopus lagopus*) and rock ptarmigan (*Lagopus muta*) (Galliformes: Phasianidae: Tetraoninae)

**DOI:** 10.1080/23802359.2017.1347834

**Published:** 2017-07-11

**Authors:** Máney Sveinsdóttir, Kristinn Pétur Magnússon

**Affiliations:** a Faculty of Natural Resource Sciences, University of Akureyri, Akureyri, Iceland;; b Collections and Systematics Department, Icelandic Institute of Natural History, Akureyri, Iceland;; c Biomedical Center, University of Iceland, Reykjavik, Iceland

**Keywords:** Mitochondrion, Illumina, next-generation sequencing, grouse, phylogeny

## Abstract

The complete mitochondrial genome sequences of the two sister species, Scandinavian willow ptarmigan *Lagopus lagopus* and Icelandic rock ptarmigan *Lagopus muta*, were characterized using next-generation sequencing. The mitogenome for willow ptarmigan was 16,677 bp long, with base composition of 30.3% A, 30.8% C, 13.3% G and 25.6% T, with a GC content of 44.1%, while for rock ptarmigan mitogenome was 16,687 bp long, with base composition of 30.2% A, 30.6% C, 13.4% G and 25.8% T, and a GC content of 44.0%. Like other Galliformes species, the mitogenomes comprised of 13 protein-coding genes, 22 tRNA, 2 rRNA and 2 non-coding regions; and control region (D-loop). All genes except *ND6* and 8 tRNA were encoded on the + strand. All protein-coding genes started with ATG, except for *COX1*, where a GTG codon was present in both willow ptarmigan and rock ptarmigan. Phylogenetic analysis of the two novel mitogenomes with other Galliformes species demonstrates close relationship within the Tetraoninae subfamily.

The two sister ptarmigan species, willow ptarmigan, *Lagopus lagopus* and rock ptarmigan *Lagopus muta*, belong to the genus *Lagopus* of the avian Tetraoninae subfamily, Phasianidae family of Galliformes. The distribution of the willow ptarmigan has an intermediate habitat preference, where it can be found in the open subalpine habitat as well as boreal forest and moorland across the Palearctic and Nearctic (Höglund et al. 2013), while the rock ptarmigan is circumpolar Holarctic, in tundra and boreal climatic zones and mountain regions (Stenkewitz et al. 2016). The subfamily Tetraoninae consists of 6 genera and 15 species, of which four mitogenomes have been published: hazel grouse (*Tetrastes bonasia*) (NC_020591) (Shen et al. 2010), Chinese grouse (*Tetrastes sewerzowi*) (NC_025318), black grouse (*Lyrurus tetrix*) (NC_024554.1) and rock ptarmigan (NC_034002.1) (Wang et al. 2017). The novel annotated mitogenomes described here of willow ptarmigan and rock ptarmigan were deposited in GenBank (accession nos. KX609784 and KX609785).

The mitogenome sequences were obtained from a whole genome, next-generation sequencing of juvenile males; willow ptarmigan from Härjedalen, Sweden, and rock ptarmigan from Mývatn, NE Iceland (accession no. LM12-040, Icelandic Institute of Natural History), and sequenced at the Illumina HiSeq^TM^ 2000 platform (San Diego, CA) at deCODE genetics, Reykjavík, Iceland, at 68× and 101× depth of coverage, respectively (Kozma et al. 2016). Sequence reads were assembled with CLC Genomics Workbench (Qiagen A/S, Aarhus, Denmark). Furthermore, Sanger sequencing (Macrogen, the Netherlands) was used to close gaps and to validate the accuracy of regions with low coverage. Automatic annotation of the mitochondrial genome was performed using MITOS WebServer (Bernt et al. 2013). DOGMA (Wyman et al. 2004) and tRNAscan-SE (Lowe and Eddy 1997) were used to further confirmation of PGCs and tRNA annotations.

The mitogenomes of the willow ptarmigan and rock ptarmigan were 16,677 bp and 16,687 bp long, respectively, and comprised of 13 protein-coding genes (PCGs), 22 tRNA, 2 rRNA and 1 control region. The base composition of the willow ptarmigan mitogenome was 30.3% A, 30.8% C, 13.3% G and T 25.6% with a GC content of 44.1%, while for rock ptarmigan 30.2% A, 30.6% C, 13.4% G and 25.8% T, and a GC content of 44.0% was observed. The total length of the 13 PCGs was 11,395 bp and 11,393 bp for willow ptarmigan and rock ptarmigan, respectively (68.3% of the mitogenome in both cases). The longest PCG was *ND5*, whereas the shortest PCG was *ATP8*. All protein-coding genes contained an ATG start codon, except for *COX1*, where instead GTG was present both in willow ptarmigan and in rock ptarmigan. A nucleotide insert in the ND3 gene, causing translational frameshifting which has been previously described in many bird species (Mindell et al. 1999), was detected in both species. The D-loop located between tRNA^Glu^ and tRNA^Phe^ genes was 1142 bp in both species and did not contain any tandem repeats. The total length of the 13 PCGs for willow ptarmigan and rock ptarmigan was 11,395 bp (68.3% of the mitogenome) and 11,393 bp (68.3% of the mitogenome). The longest PCG was *ND5*, whereas the shortest PCG was *ATP8*.

For phylogenetic analysis, we subjected 17 complete mitogenomes, for MAFFT alignment (version 7) (Katoh and Standley 2013) at EMBL-EBI web, to use in maximum-likelihood phylogeny analysis in CLC Genomics Workbench (version 10.0.3), using UPGMA and Jukes–Cantor algorithms, replicated 1000 times (Figure 1). For the analysis, we included the two novel mitogenomes described in this paper, together with 14 published mitogenomes of 13 other species of Galliformes (Valverde et al. 1994; Shen et al. 2010; Liu et al. 2014; Li et al. 2016; Sveinsdóttir et al. 2017; Wang et al. 2017) using *Branta canadensis* (NC_007011.1) as an outgroup. The phylogeny shows clearly the avian order of Galliformes (chickens, turkey, quail and allies) and how the five Tetraoninae subfamily species cluster accordingly. Further, the genetic distance between the two *Lagopus muta* birds, from Magadanskaya Oblast, East Russia, and from Iceland may indicate a long substantial time of separation. The characterized mitogenomes of two *Lagopus* species will add to our understanding of the evolution of Galliformes species and will be useful in population genetics and phylogenetics (Galtier et al. 2009).

**Figure 1.**
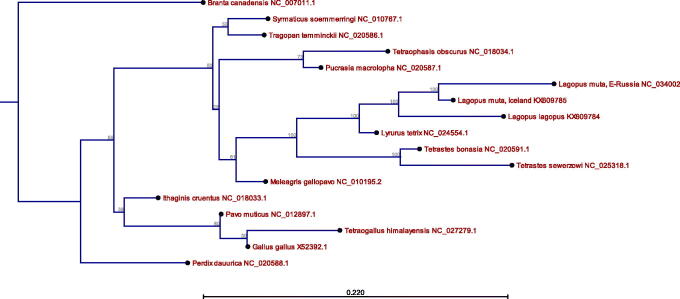
Maximum-likelihood (ML) phylogenetic tree of *L. lagopus*, *L. muta* and 14 other Galliformes mitogenomes with *Branta canadensis* as an outgroup. Accession numbers are listed behind the species names. The numbers by each node indicate the ML bootstrap support values. Scale shows evolutionary distance.
